# Opportunities for Improving Antimicrobial Stewardship: Findings From a Prospective, Multi-Center Study in Three Low- or Middle-Income Countries

**DOI:** 10.3389/fpubh.2022.848802

**Published:** 2022-04-25

**Authors:** Florida J. Muro, Furaha S. Lyamuya, Charles Kwobah, John Bollinger, Champica K. Bodinayake, Ajith Nagahawatte, Bhagya Piyasiri, Ruvini Kurukulasooriya, Shamim Ali, Rose Mallya, Robert Rolfe, Anushka Ruwanpathirana, Tianchen Sheng, Truls Østbye, Richard Drew, Peter Kussin, Christopher W. Woods, Deverick J. Anderson, Blandina T. Mmbaga, L. Gayani Tillekeratne

**Affiliations:** ^1^Community Health Department, Kilimanjaro Christian Medical Centre (KCMC), Moshi, Tanzania; ^2^Institute of Public Health, Kilimanjaro Christian Medical University College (KCMUCo), Moshi, Tanzania; ^3^Kilimanjaro Christian Research Institute, Moshi, Tanzania; ^4^Internal Medicine Department, Kilimanjaro Christian Medical Centre (KCMC), Moshi, Tanzania; ^5^Moi University/Moi Teaching and Referral Hospital, Eldoret, Kenya; ^6^Duke-Margolis Center for Health Policy, Washington, DC, United States; ^7^Duke Global Health Institute, Durham, NC, United States; ^8^Duke University, Durham, NC, United States; ^9^University of Ruhuna, Galle, Sri Lanka; ^10^Teaching Hospital Karapitiya, Galle, Sri Lanka; ^11^Reproductive and Child Health, Kilimanjaro Christian Medical Centre, Moshi, Tanzania; ^12^Ohio State University, Columbus, OH, United States; ^13^Campbell University College of Pharmacy and Health Sciences, Buies Creek, NC, United States; ^14^Duke Center for Antimicrobial Stewardship and Infection Prevention, Durham, NC, United States; ^15^Paediatric and Child Health Department, Kilimanjaro Christian Medical Centre, Kilimanjaro Christian Medical University College, Moshi, Tanzania

**Keywords:** antimicrobial stewardship, antimicrobial agents, less developed countries (LDCs), antimicrobial resistance (AMR), respiratory tract infection (RTI)

## Abstract

**Background:**

To develop effective antimicrobial stewardship programs (ASPs) for low- and middle-income countries (LMICs), it is important to identify key targets for improving antimicrobial use. We sought to systematically describe the prevalence and patterns of antimicrobial use in three LMIC hospitals.

**Methods:**

Consecutive patients admitted to the adult medical wards in three tertiary care hospitals in Tanzania, Kenya, and Sri Lanka were enrolled in 2018–2019. The medical record was reviewed for clinical information including type and duration of antimicrobials prescribed, indications for antimicrobial use, and microbiologic testing ordered.

**Results:**

A total of 3,149 patients were enrolled during the study period: 1,103 from Tanzania, 750 from Kenya, and 1,296 from Sri Lanka. The majority of patients were male (1,783, 56.6% overall) with a median age of 55 years (IQR 38–68). Of enrolled patients, 1,573 (50.0%) received antimicrobials during their hospital stay: 35.4% in Tanzania, 56.5% in Kenya, and 58.6% in Sri Lanka. At each site, the most common indication for antimicrobial use was lower respiratory tract infection (LRTI; 40.2%). However, 61.0% received antimicrobials for LRTI in the absence of LRTI signs on chest radiography. Among patients receiving antimicrobials, tools to guide antimicrobial use were under-utilized: microbiologic cultures in 12.0% and microbiology consultation in 6.5%.

**Conclusion:**

Antimicrobials were used in a substantial proportion of patients at tertiary care hospitals across three LMIC sites. Future ASP efforts should include improving LRTI diagnosis and treatment, developing antibiograms to direct empiric antimicrobial use, and increasing use of microbiologic tests.

## Introduction

Antimicrobial resistance (AMR) is one of the greatest threats to health, and without the prioritization of rational use of antimicrobials, it is estimated that AMR may cause up to 10 million deaths annually by 2050 (1). Inappropriate use of antimicrobials increases selection pressure, leading to the emergence of resistance (2). Among hospitalized patients, unnecessary antimicrobial prescription, inappropriate dosages, and inadequate monitoring have all been shown to be facets of inappropriate use of antimicrobials (3). In addition, the lack of local treatment guidelines and the lack of microbiologic data can drive inappropriate antimicrobial use (4). Low- and middle-income countries (LMICs) in Sub-Saharan Africa and Asia carry the greatest burden of infectious diseases (5), and thus have a higher potential for inappropriate antimicrobial use.

Given the urgent need to conserve limited antimicrobials, many nations have adopted the Global Action Plan on Antimicrobial Resistance developed by the World Health Organization (WHO), and member states have also developed National Action Plans for AMR (6). Locally, hospitals are starting to develop and implement antimicrobial stewardship programs (ASPs) that monitor and improve antimicrobial use for better patient outcomes. ASPs have been shown to improve the appropriate use of antimicrobials, especially in high-income countries (7–10). However, ASPs are less widely implemented in LMICs (11, 12). A few studies in better-resourced LMICs such as South Africa have shown that a network of ASPs can be implemented successfully (13, 14). At the international level, stakeholders have called for the need for standardized resources and guidelines for ASPs in LMICs, as well as the assessment of the cost effectiveness of such programs (15, 16).

To improve the effectiveness of ASPs in LMIC settings, it is important to determine key targets for improving antimicrobial use. Our team has been working to support the implementation of ASPs in a context-sensitive manner in Tanzania, Kenya, and Sri Lanka. Our US Academic Medical Center has strong research relationships with tertiary care hospitals in each of these countries. As these are the centers where we are actively working, we felt it would be appropriate to determine the prevalence and patterns of antimicrobial use that were shared between or unique to these tertiary care centers in three LMICs. We previously reported findings from a parallel qualitative study using interviews with physicians to identify barriers to ASP implementation (17). The aim of this study was to quantify the prevalence and patterns of antimicrobial use at these same sites, such that we could identify broad targets for improving antimicrobial use.

## Materials and Methods

### Setting

This study was conducted at three tertiary care centers in Tanzania, Kenya, and Sri Lanka.

The first is a 630-bed zonal referral level facility located in northeastern Tanzania. The hospital has four adult medical wards (2 male, 2 female) which admit patients ≥ 14 years of age. Patients pay for clinical care, diagnostic testing, radiographs, and medications. The facility has an infectious diseases consult service, which provides advice on infectious disease-related cases on request and is staffed by an attending-level physician. There is a nascent ASP at the hospital that was formed in July 2017 by the hospital administration. This team includes representatives from clinical departments, pharmacy and the clinical laboratory who are tasked with formulating measures and policies to govern the rational use of antimicrobials. Pharmacists dispense antimicrobials and other drugs, but do not provide advice on therapy.

The second is a 990-bed public facility located in western Kenya. The hospital has two adult medical wards (1 male, 1 female) which admit patients who are ≥14 years of age. Patients pay for clinical care, diagnostic testing, radiographs, and medications. The facility did not have an infectious disease or microbiology consult service or a formal ASP at the time of the study. Clinical pharmacists participate in ward rounds with the clinicians and provide advice on therapy.

The third facility in southern Sri Lanka is a 1,550-bed public, tertiary care center that provides all clinical care, diagnostic testing and radiographs, and medications free of charge to patients. The hospital has 10 adult medical wards (5 male, 5 female) which admit patients who are ≥12 years of age. A microbiology consult service is staffed by an attending-level physician who is trained in microbiology and is available on request to provide advice on infectious disease-related cases. Pharmacists dispense antimicrobials and other drugs but do not provide advice on therapy. The hospital did not have a formal ASP at the time of study.

### Study Population and Procedures

All patients admitted to the adult medical wards were eligible for enrollment by trained research assistants, who were qualified nurses. Enrollments were conducted for at least 5 months at each site: from June 2018 to March 2019 in Tanzania, December 2018 to April 2019 in Kenya, and February 2018 to December 2018 in Sri Lanka. All patients admitted within the prior 24–48 h were eligible for enrollment, and ~8–15 patients were enrolled daily. If the number of patients eligible per day was greater than possible to enroll based on available research personnel, a systematic random sample was selected—i.e., every 2nd or 3rd eligible patient using a pre-determined sampling strategy of each hospital's wards to reduce selection bias.

All sociodemographic and clinical data were obtained from the medical records; there was no direct contact between the research team and patients. Information regarding types of antimicrobials prescribed, indications for antimicrobials, and duration and outcome of hospitalization were collected from the medical record. Types of antimicrobials captured included anti-bacterials, anti-virals, anti-fungals, anti-parasitics, and anti-tuberculosis medications given in either oral or intravenous form. Indications for antimicrobial use were extracted as documented in the medical record, or if such documentation was missing, were inferred based on documented clinical information. If an indication could not be reliably inferred by the trained research assistant, they had the option of marking “fever only, no other indication,” “leukocytosis only, no other indication,” or “unknown.” Up to two indications could be marked for a patient during their hospitalization (i.e., if a patient had two separate infections such as bacterial endocarditis and a catheter-associated urinary tract infection, which may have been concurrent or sequential during the hospitalization) or if the type of infection was not clear to the treating physician (i.e., infectious syndrome could be pyelonephritis or diverticulitis based on clinical symptoms and data available, and patient received antimicrobial therapy that would be effective against both conditions). Utilization of tools to guide antimicrobial use, including microbiologic culture data, a creatinine test to potentially adjust antimicrobial dosing, and consultation of microbiology/infectious diseases services, were also recorded from the medical chart.

In September 2018 (7 months into the study), the physicians in Sri Lanka had an educational session that included a didactic session on the importance of antimicrobial stewardship and the diagnosis and treatment of lower respiratory tract infection (LRTI). A reminder card with treatment algorithms for LRTI based on national treatment guidelines was handed out to physicians. In Tanzania, didactic sessions on the importance of antimicrobial stewardship and the diagnosis and treatment of LRTI were held in November 2018 (5 months into the study). No educational interventions were conducted in Kenya.

### Data Analysis

All data were collected manually on paper questionnaires entered into a Research Electronic Data Capture (REDCap) database. Overall prevalence of antimicrobial use was calculated by dividing the number of patient receiving one or more antimicrobial agent during the study by the total number of patients. All statistical analyses were conducted using R version 3.6.3 (R Foundation for Statistical Computing, Vienna, Austria). The χ^2^ test was used to compare antimicrobial use prevalence between sites and features associated with antimicrobial use overall, with a *p*-value < 0.05 indicating statistical significance.

#### Appropriateness of Antimicrobials

Potentially redundant combinations of antimicrobials were defined as the concurrent use of ≥2 beta-lactam antibiotics or ≥2 antibiotics active against anaerobes, *Pseudomonas aeruginosa*, or methicillin-resistant *Staphylococcus aureus*. The appropriateness of antimicrobials for two indications was also determined: LRTI and urinary tract infection (UTI). Appropriateness was assessed for these conditions because they were the two most common indications for antimicrobial use at these hospitals overall, and since anecdotal experience suggested that inappropriate use for these conditions may be common. Antimicrobials for LRTI were considered potentially inappropriate if the patient did not have radiographic signs consistent with LRTI prior to antimicrobial initiation, with radiographs read by either a treating physician or radiologist at the hospital (18). Patients with chronic obstructive pulmonary disease (COPD) were excluded from this analysis since they may benefit from antimicrobial therapy despite having no radiographic signs of LRTI. Patients without chest x-rays or chest CTs were also excluded from this analysis, since they may have been unable to pay for these imaging modalities and the treating physician may have had to make empiric treatment decisions. Antimicrobials were considered potentially inappropriate for UTI if the patient was treated for asymptomatic bacteriuria in the absence of being pregnant or having an upcoming invasive genito-urinary procedure (19). Asymptomatic bacteriuria was defined as presence of bacteriuria without at least one symptom consistent with UTI. Symptoms included fever not explained by another cause, dysuria, frequency, urgency, suprapubic pain, costovertebral angle tenderness, and nausea/vomiting among patients without a catheter. Symptoms included fever not explained by another cause, suprapubic pain, costovertebral angle tenderness, or nausea/vomiting in patients with a urinary catheter at onset of symptoms or within prior 24 h. All information regarding symptoms and exam findings was gathered from the medical record.

### Ethics

Ethical approval for this study was obtained from the Kilimanjaro Christian Medical College Research Ethics and Review Committee and the National Institute for Medical Research (Tanzania), the Institutional Research and Ethics Committee of Moi University/Moi Teaching and Referral Hospital (Kenya), AMPATH (Kenya), the Ethical Review Committee of the Faculty of Medicine, University of Ruhuna (Sri Lanka), and the Duke University Institutional Review Board (United States). Permission to access and review patients' medical records was also sought from the facility directors and physicians in charge of the wards.

## Results

### Demographic and Clinical Characteristics

Across the three sites, a total of 3,149 patients were enrolled: 1,103 from Tanzania, 750 from Kenya, and 1,296 from Sri Lanka (Table 1). The majority of patients at each site were male (1,783, 56.6% overall) with a median age of 55 years (IQR 38–68) and a median hospitalization duration of 3–5 days across sites (4 days overall, IQR 2–7). Of enrolled patients, 2,411 (76.6%) had one or more chronic medical conditions. Common chronic medical conditions included hypertension (989, 31.4%), diabetes mellitus (649, 20.6%), malignancy (343, 10.9%), chronic kidney disease (270, 8.6%), and ischemic heart disease (246, 7.8%). Hypertension was the most common chronic medical condition in Tanzania (31.8%) and Sri Lanka (40.6%), while malignancy was the most common chronic medical condition in Kenya (32.9%). HIV prevalence was 9.2% in Tanzania, 15.7% in Kenya, and 0.1% in Sri Lanka. Mortality during hospitalization varied across the three sites, with 22.8% in Tanzania, 21.6% in Kenya, and 1.4% in Sri Lanka.

**Table 1:** Demographic characteristics, site-specific antimicrobial use, and outcomes among patients at three tertiary care hospitals in Tanzania, Kenya, and Sri Lanka (*N* = 3,149).

**Characteristic**			**Country**			
			**Tanzania**	**Kenya**	**Sri Lanka**	**Overall**
			**(*n* = 1,103)**	**(*n* = 750)**	**(*n* = 1,296)**	**(*n* = 3,149)**
**Demographic and clinical information**						
Age (years)^a^	<18		26 (2.4)	28 (3.7)	35 (2.7)	89 (2.8)
	18–45		360 (32.6)	329 (43.9)	322 (24.8)	1011 (32.1)
	46–65		370 (33.5)	224 (29.9)	511 (39.4)	1105 (35.1)
	>65		345 (31.3)	169 (22.5)	419 (32.3)	933 (29.6)
Sex	Male		668 (60.6)	390 (52.0)	725 (55.9)	1783 (56.6)
Chronic medical conditions	Hypertension		351 (31.8)	112 (14.9)	526 (40.6)	989 (31.4)
	Diabetes mellitus		171 (15.5)	62 (8.3)	418 (32.2)	649 (20.6)
	Chronic liver disease		27 (2.4)	10 (1.3)	21 (1.6)	49 (1.6)
	Chronic kidney disease		80 (7.2)	89 (11.9)	102 (7.9)	270 (8.6)
	Asthma		21 (1.9)	3 (0.4)	218 (16.8)	242 (7.7)
	Heart failure		112 (10.2)	69 (9.2)	10 (0.8)	180 (5.7)
	Ischemic heart disease		1 (0.1)	8 (1.1)	237 (18.3)	246 (7.8)
	COPD^b^/emphysema		19 (1.7)	35 (4.7)	71 (5.5)	125 (4.0)
	Malignancy		117 (10.6)	247 (32.9)	4 (0.4)	343 (10.9)
	HIV^c^		101 (9.2)	118 (15.7)	1 (0.1)	218 (6.9)
	Hyperlipidemia		0	0	89 (6.9)	89 (2.8)
	Psychiatric disorder		3 (0.3)	3 (0.4)	10 (0.8)	16 (0.5)
	Hematologic disorder		8 (0.7)	15 (2.0)	21 (1.6)	44 (1.4)
	Alcohol abuse		0	1 (0.1)	5 (0.4)	6 (0.2)
	Neurologic disorder		14 (1.3)	18 (2.4)	73 (5.6)	105 (3.3)
	Other chronic lung disease		0	0	13 (1.0)	13 (0.4)
	Other immunosuppressed disease^d^		2 (0.2)	43 (5.7)	5 (0.4)	50 (1.6)
	Other diseases		64 (5.8)	52 (6.9)	233 (18.0)	349 (11.1)
**Measures associated with antimicrobial use and clinical outcomes**						
Antimicrobial allergy	Not documented		497 (45.0)	43 (5.7)	683 (52.7)	1123 (38.8)
	Documented	Allergy exists	13 (1.2)	5 (0.7)	41 (3.2)	59 (1.9)
		No allergy	593 (53.8)	702 (93.6)	572 (44.1)	1867 (59.3)
Antimicrobial therapy received			390 (35.4)	424 (56.5)	759 (58.6)	1573 (50.0)
Hospitalization duration (days)			5 (3–8)	5 (3–9)	3 (2–4)	4 (2–7)
Clinical status at hospital discharge or transfer	Alive		845 (76.6)	583 (77.7)	1267 (97.8)	2695 (85.6)
	Dead		251 (22.8)	162 (21.6)	19 (1.4)	432 (13.7)
	Missing		7 (0.6)	5 (0.7)	10 (0.8)	22 (0.7)

a *There are 11 missing age, with 2 in Tazania and 9 in Sri Lanka*.

b *COPD is chronic obstructive pulmonary disease*.

c *HIV included in analyses for both chronic medical condition and indication for antimicrobial use*.

d *Other immunosuppressed diseases include acute lymphoblastic leukemia, acute myelogenous leukemia, aplastic anemia, bicytopenia, chronic myelogenous leukemia, idiopathic thrombocytopenic purpura, unspecified lymphoma, multiple myeloma, non-hodgkin's lymphoma, Parkinson disease, pneumocystis pneumonia, pemphigus, progressive multifocal leukoencephalopathy, systemic lupus erythematosus, chronic lymphocytic leukemia, mycosis fungoides, and refractory cytopenia with multilineage dysplasia*.

### Antimicrobial Use and Indications

Overall, 1,573 (50.0%) of enrolled patients received antimicrobials during hospitalization. The prevalence of antimicrobial use was significantly different between sites: 58.6, 35.4, and 56.5% in Sri Lanka, Tanzania, and Kenya, respectively (*p* ≤ 0.001). Among patients for whom an antimicrobial was prescribed, the leading comorbid conditions were hypertension (471, 29.9%), diabetes mellitus (320, 20.3%), asthma (164, 10.4%), chronic kidney disease (124, 7.9%), and malignancy (116, 7.4%). HIV was present in 161 (10.2%) of patients receiving antimicrobials. When excluding HIV, having a comorbid condition was associated with less antimicrobial use (47.0% of patients with a comorbid condition received antimicrobials vs. 56.4% without a comorbid condition, *p* < 0.001).

The most commonly used antimicrobials were third-generation cephalosporins (821, 52.2%), amoxicillin-clavulanic acid (434, 27.6%), macrolides (293, 18.6%), and metronidazole (287, 18.2%) (Table 2). In Tanzania and Kenya, third-generation cephalosporins were most common (73.3 and 72.9% of patients receiving antimicrobials, respectively), while in Sri Lanka, amoxicillin-clavulanate was most common (49.8%). Only 9.4 and 4.0% of patients overall were prescribed fluoroquinolones or carbapenems, respectively. Of patients receiving antimicrobials, most patients received either one antimicrobial agent (751, 47.7%) or two antimicrobial agents (587, 37.3%) during hospitalization. The median duration of antimicrobial use across sites was 4–6 days (4 days overall, IQR 3–7 days). The most common indications for antimicrobial use included LRTI in 633 (40.2%), UTI in 154 (9.8%), skin and soft tissue infection (SSTI) in 91 (5.8%), and tuberculosis in 83 (5.3%) patients. At each site, LRTI was the most common indication for antimicrobial use (50.0% in Tanzania, 30.0% in Kenya, and 41.0% in Sri Lanka).

**Table 2:** Antimicrobial use, indications, and tools to guide therapy across study sites in Tanzania, Kenya, and Sri Lanka (*N* = 1,573).

**Antimicrobial therapy**		**Country**			
		**Tanzania (*n* = 390)**	**Kenya (*n* = 424)**	**Sri Lanka (*n* = 759)**	**Overall (*n* = 1,573)**
Antimicrobial	Narrow-spectrum penicillin^a^	55 (14.1)	13 (3.1)	80 (10.5)	148 (9.4)
	Vancomycin	2 (0.5)	19 (4.5)	5 (0.6)	26 (1.6)
	1st and 2nd generation cephalosporin	0	8 (1.9)	13 (1.7)	21 (1.3)
	3rd generation cephalosporin	286 (73.3)	309 (72.9)	226 (29.8)	821 (52.2)
	4th generation cephalosporin	0	31 (7.3)	2 (0.3)	33 (2.1)
	Amoxicillin and clavulanic acid	24 (6.2)	32 (7.5)	378 (49.8)	434 (27.6)
	Metronidazole	158 (40.5)	86 (20.3)	43 (5.7)	287 (18.2)
	Macrolides^b^	18 (4.6)	102 (24.0)	173 (22.8)	293 (18.6)
	Tetracycline/doxycycline	1 (0.2)	1 (0.2)	59 (7.8)	61 (3.9)
	Fluoroquinolone	13 (3.3)	35 (8.2)	100 (13.2)	148 (9.4)
	Aminoglycoside	7 (1.8)	2 (0.5)	7 (0.9)	16 (1.0)
	Carbapenem	2 (0.5)	21 (5.0)	40 (5.3)	63 (4.0)
	Anti-tuberculosis therapy	12 (3.1)	46 (10.8)	0	58 (3.7)
	Antifungal azole	8 (2.0)	41 (9.7)	0	49 (3.1)
	Amphotericin	0	6 (1.4)	0	6 (0.4)
	Oseltamivir	0	0	0	0
	Acyclovir	0	5 (1.2)	5 (0.6)	10 (0.6)
	Antimalarial^c^	0	7 (1.6)	0	7 (0.4)
	Other intravenous/oral medication^d^	45 (11.5)	65 (15.3)	91 (12.0)	201 (12.8)
	Anti-retrovirals	5 (1.3)	22 (5.2)	0	27 (1.7)
Number of antimicrobials	1	179 (45.9)	149 (35.1)	423 (55.7)	751 (47.7)
	2	172 (44.1)	162 (38.2)	253 (33.3)	587 (37.3)
	3	25 (6.4)	477 (11.1)	62 (8.2)	134 (8.5)
	4 or more	14 (3.6)	66 (15.6)	21 (2.8)	101 (6.4)
Indication for antimicrobial use	Upper respiratory tract infection	3 (0.8)	1 (0.2)	54 (6.7)	58 (3.7)
	Lower respiratory tract infection	195 (50.0)	127 (30.0)	311 (41.0)	633 (40.2)
	Tuberculosis	30 (7.7)	46 (10.8)	7 (0.9)	83 (5.3)
	Meningitis/encephalitis	10 (2.6)	22 (5.2)	11 (1.4)	43 (2.7)
	Skin and soft tissue infection	23 (5.9)	8 (1.9)	60 (7.9)	91 (5.8)
	Gastroenteritis	16 (4.1)	19 (4.5)	41 (5.4)	76 (4.8)
	Urinary tract infection^e^	15 (3.8)	24 (5.7)	115 (15.2)	154 (9.8)
	Endocarditis	0	3 (0.7)	3 (0.4)	6 (0.4)
	Leptospirosis	0	0	49 (6.4)	49 (3.1)
	Malaria	0	7 (1.6)	0	7 (0.4)
	Surgical prophylaxis	1 (0.2)	0	5 (0.6)	6 (0.4)
	Medical prophylaxis	3 (0.8)	14 (3.3)	0	17 (1.1)
	Bacteremia	2 (0.5)	2 (0.5)	0	4 (0.2)
	Fever only (no other indication)	8 (2.0)	0	3 (0.4)	11 (0.7)
	Other	42 (10.8)	67 (15.8)	39 (5.1)	148 (9.4)
	No clear indication	41 (10.5)	140 (33.0)	85 (11.2)	266 (16.9)
Duration of therapy (days)	6 (4–8)	5 (3–9)	4 (3–5)	4 (3–7)	
Microbiology/infectious diseases consulted or provided input	Yes	92 (23.6)	2 (0.5)	8 (1.1)	102 (6.5)
	No	107 (27.4)	193 (45.5)	735 (96.8)	1035 (65.8)
	Service not available	0	226 (53.3)	0	226 (14.4)
	Missing	191 (49.0)	3 (0.7)	16 (2.1)	210 (13.4)
Creatinine obtained before or on same day starting antimicrobial use^f^	Yes	249 (63.8)	212 (50.0)	406 (53.5)	867 (55.1)
	No	34 (8.7)	100 (23.6)	247 (32.5)	381 (24.2)
	Missing	7 (0.6)	5 (0.7)	10 (0.8)	22 (0.7)
Culture result	Yes	29 (7.4)	33 (7.8)	127 (16.7)	189 (12.0)
	No	239 (61.3)	386 (91.0)	588 (77.5)	1213 (77.1)
	Missing	122 (31.3)	5 (1.2)	44 (5.8)	171 (10.9)
Discharged/ transferred on antimicrobials	59 (15.1)	148 (34.9)	132(17.4)	339 (21.6)	

a *Narrow-spectrum penicillin includes amoxicillin, ampicillin, dicloxacillin, nafcillin, oxacillin, or cloxacillin*.

b *Macrolides include erythromycin, azithromycin, or clarithromycin*.

c *Antimalarial drugs include artemether-lumefantrine, artesunate, or quinine*.

d *Other antimicrobials include trimethoprim and sulfamethoxazole, clindamycin, piperacillin/tazobactam, ampicillin/cloxacillin, albendazole, tinidazole, nitrofurantoin, and piperacillin*.

e *Urinary tract infection includes cystitis and pyelonephritis*.

f *Missing means no record of either antimicrobial therapy date or creatinine obtained date*.

Antimicrobial use was associated with death in this cohort (17.5% mortality in those who received antimicrobials vs. 10.0% in those who did not, *p* < 0.001). The most common reasons for antimicrobial use among patients who died were LRTI 125 (45.4%), tuberculosis 28 (10.2%), UTI 13 (4.7%), and meningitis/encephalitis 19 (6.9%).

### Tools for Guiding Antimicrobial Use

Among patients who received antimicrobial therapy, 189 (12.0%) had microbiologic culture data relevant to their antimicrobial use indication and 867 (55.1%) had a documented creatinine level before or on the same day as starting antimicrobial therapy. Availability of microbiologic data across the three sites was similar: 29 (7.4%) among patients receiving antimicrobials in Tanzania, 33 (7.8%) in Kenya, and 127 (16.7%) in Sri Lanka. Of microbiologic culture data, 26 (15.7%) were sputum cultures, 46 (27.9%) were blood cultures and 93 (56.4%) were urine cultures. Overall, 83 (43.9%) of the cultures were positive for an organism. Obtaining a creatinine level was also similar across sites: 867 (55.1%) in Tanzania, 249 (63.8%) in Kenya, and 406 (53.5%) in Sri Lanka. Microbiology/ infectious diseases specialty services were consulted for input on antimicrobial use in 100 (6.4%) of study patients, with 92 (23.6%) in Tanzania and 8 (1.0%) in Sri Lanka (the Kenyan site does not have this service).

### Appropriateness of Antimicrobial Use

Of the 1,573 patient who received antimicrobials, 83 (5.3%) received double beta-lactam coverage and 21 (1.3%) received double anaerobic coverage therapy (Table 3). The proportion of patients receiving double beta-lactam or double anaerobic coverage was similar across sites.

**Table 3:** Appropriateness of antimicrobial therapy across study sites in Tanzania, Kenya, and Sri Lanka.

**Redundant antimicrobial therapy**				
**Characteristic**	**Country**			
	**Tanzania (*n* = 390)**	**Kenya** **(*n* = 424)**	**Sri Lanka (*n* = 759)**	**Overall** **(*n* = 1,573)**
Double beta-lactam coverage	11 (2.8)	21 (5.0)	51 (6.7)	83 (5.3)
Double anaerobic coverage	1 (0.2)	8 (1.9)	12 (1.6)	21 (1.3)
**LRTI**^a^ **as the indication for antimicrobial therapy**				
**Characteristic**	**Country**			
	**Tanzania (*n* = 185)**	**Kenya** **(*n* = 104)**	**Sri Lanka (*n* = 265)**	**Overall** **(*n* = 554)**
Patient has radiographic signs of pneumonia within 48 h of starting antibiotics	116 (62.7)	65 (62.5)	35 (13.2)	216 (39.0)
Sputum culture obtained	0	2 (1.9)	18 (6.8)	20 (3.6)
**UTI**^b^ **as the indication for antimicrobial therapy**				
**Characteristic**	**Country**			
	**Tanzania (*n* = 16)**	**Kenya** **(*n* = 23)**	**Sri Lanka (*n* = 112)**	**Overall** **(*n* = 151)**
Symptoms present before initiation of treatment	9 (56.2)	19 (82.6)	106 (94.6)	134 (88.7)
Fever (not explained by another cause)	6 (37.5)	2 (8.7)	99 (88.4)	107 (70.9)
Suprapubic pain	2 (12.5)	2 (8.7)	69 (61.6)	73 (48.3)
Dysuria	0	4 (17.4)	58 (51.8)	62 (41.0)
CVA^c^ tenderness	0	4 (17.4)	2 (1.8)	6 (3.9)
Frequency	0	0	40 (35.7)	40 (26.5)
Nausea/vomiting	3 (18.8)	0	63 (56.2)	66 (43.7)
Urgency	1 (6.2)	0	0	1 (0.7)
Urine culture	5 (31.2)	3 (13.0)	56 (50.0)	64 (42.4)

a *LRTI is lower respiratory tract infections*.

b *UTI is urinary tract infection*.

c *CVA is flank or costovertebral angle*.

When considering the 554 patients with LRTI as the indication for antimicrobial therapy and excluding those with COPD as a chronic medical condition, 216 (39.0%) had radiographic signs of pneumonia within 48 h of initiating antimicrobial treatment (Table 3). This proportion varied widely across sites with 116 (62.7%) in Tanzania, 65 (62.5%) in Kenya, and only 35 (13.2%) in Sri Lanka. The use of sputum cultures was generally low across sites: 0 (0%) in Tanzania, 2 (1.9%) in Kenya, and 18 (6.8%) in Sri Lanka.

When considering the 151 patients with UTI as the indication for antimicrobial therapy and excluding those who were pregnant or had an upcoming genito-urinary procedure, 134 (88.7%) had symptoms present before the initiation of antimicrobials (Table 3). The proportion with symptoms varied across sites with 9 (56.2%), 19 (82.6%), and 106 (94.6%) with symptoms before treatment in Tanzania, Kenya, and Sri Lanka, respectively. A total of 64 (42.4%) of patients with UTI had a urine culture performed. The proportion with urine cultures again varied across the three sites, with 5 (31.2%), 3 (13.0%), and 56 (50.0%) patients having a urine culture performed in Tanzania, Kenya, and Sri Lanka, respectively.

## Discussion

In this study, we describe the prevalence and patterns of antimicrobial use at tertiary-level hospitals in three lower middle-income countries: Tanzania, Kenya, and Sri Lanka. Despite distinct disease epidemiology, patient populations, and health systems across the sites, common themes emerged regarding antimicrobial use. First, a substantial proportion (35- 59%) of admitted patients were prescribed antimicrobials at each site, with most patients receiving broad-spectrum antimicrobials. Second, the most common indication for antimicrobial use was LRTI at each hospital. Diagnosis was made mostly on clinical grounds, with only a minority of patients treated for LRTI having radiographic evidence of pneumonia. Finally, the treatment of LRTI and other infectious syndromes was mostly empiric, with <15% having an associated microbiologic culture. Our findings suggest that there are basic, common actions that could be undertaken by ASPs at these and similar sites in lower-resource settings to improve antimicrobial use.

Similar studies have reported high levels of antimicrobial use in these three countries. In a point-prevalence survey of antimicrobial use of inpatients in six referral hospitals in Tanzania in 2019, 62.3% of patients were prescribed antimicrobials (20). Similarly, among hospitalized patients across 14 public hospitals in Kenya in 2018, 46.7% received antimicrobial prescriptions (21). A point-prevalence study among patients hospitalized in 5 public hospitals in Sri Lanka in 2017 showed that 54.6% received antimicrobial prescriptions (22). The Tanzanian study used WHO point prevalence survey (PPS) methodology to estimate the prevalence of antibiotic use while the Kenyan study used the web-based Global Point Prevalence Survey of Antimicrobial Consumption and Resistance (GLOBAL-PPS) tool (23, 24). The Sri Lankan study employed a point-prevalence survey with repeated enrollments to attain sample size. Most of the hospitals included were referral level facilities and public. Observed differences across sites may be due to differences in the patient populations enrolled, disease epidemiology, or seasonal differences in antimicrobial prescribing. Further, differences may be driven by varying local antimicrobial use guidelines or policies and their implementation, as well as differences in accessibility of antimicrobials. We found that antimicrobial use was associated with mortality in our cohort, which may indicate the underlying severity of illness in these patients or that antimicrobials were not being used effectively in this subset of patients. Further studies exploring the causes of death and treatments received are needed.

Despite different geography and disease epidemiology across sites, LRTI was the most common indication for antimicrobials, including among those who died. However, chest radiography findings consistent with LRTI were not often present, and few patients received sputum cultures. Interventions focusing on the diagnosis and management of LRTI should be a major target of ASPs at each of these study sites, and likely in similar settings across the globe. LRTI is a common reason for seeking medical care and the leading infectious cause of death globally (25–27). The diagnosis of LRTI is hampered by limitations in existing respiratory diagnostics such as sputum culture and multiplex polymerase chain reaction testing of nasopharyngeal samples, which may have variable sensitivity, are limited to a few organisms, and do not distinguish colonization from infection (28). Nonetheless, our findings suggest that even existing diagnostics are used suboptimally at these sites, with <5% of patients with LRTI receiving sputum cultures. The diagnosis of LRTI in the absence of radiographic findings may suggest misdiagnosis, which is especially concerning since LRTI was the most common indication for antimicrobial use among patients who died. Further, while sputum cultures may take several days to result and thus may not be as useful in guiding antimicrobial therapy, a sputum Gram stain alone may provide valuable information regarding the presence of important pathogens such as *Streptococcus pneumoniae* (29).

As with LRTI, the treatment of other infectious syndromes should also generally be guided by culture and antimicrobial susceptibility data in concert with treatment guidelines and clinical judgment. However, the use of microbiological data in this study was quite low. Studies show that clinical microbiological services in low-resource settings are traditionally limited due to a multitude of reasons including lack of infrastructure, equipment, quality assurance, and personnel and training (30). However, all hospitals included in this study were tertiary care hospitals and had the capacity to perform antimicrobial susceptibility testing. Therefore, the low use of microbiological testing suggests that clinicians may not appreciate the value of microbiological testing or may have had decreased access to testing at the time of the study. Results from our parallel qualitative study at these same sites suggested that clinicians were at times concerned about the reliability of culture results, with concerns that cultures may be contaminated, negative even when there was high suspicion of bacterial infection, or may take a long time to result. These results suggest that improved education of clinicians regarding the use of microbiological data, greater partnership and communication between clinicians and the microbiology laboratory, and improved access to microbiological diagnostics may all be useful in these resource-limited settings (31). Limited microbiological capacity as well as a lack of willingness to seek microbiological data negatively affect ASP implementation and subsequently harm efforts to contain antimicrobial resistance. In settings where microbiologic data may be limited, local antibiograms can guide empiric treatment decisions (32). For our sites, developing and implementing antibiograms should be high priorities for improving antimicrobial use and the treatment of infectious syndromes.

Finally, we identified other potential targets for improving antimicrobial use that were common across these three sites. The indication for antimicrobial use in almost 20% of patients was unclear, based on review of the medical records. In addition to improved access to diagnostics, proper documentation for an indication of antimicrobial could curb antimicrobial overuse and as a result improve its prescribing (33). Microbiology/infectious diseases specialty services were consulted in <10% of cases, and could be consulted more frequently for guidance in antimicrobial use. We also found that checking a creatinine level, which is a relatively low-cost intervention, was only obtained in ~55% of patients receiving antimicrobials and could be improved to adjust antimicrobial dosing and to minimize patient harm (34). These findings suggest potential targets for ASPs at these hospitals that are common across these LMIC sites.

Some limitations in this study must be noted. Our findings were collected from tertiary care hospitals in three LMICs, and may not be generalizable to hospitals at other levels of care or in other LMICs. However, despite differences in geography and epidemiology, we were able to identify ASP targets that were common across these sites: documentation of indication for antimicrobial prescription, use of microbiologic culture data, partnership and consultation with microbiology/infectious diseases services, and appropriate diagnosis and treatment of LRTI. Data collection was conducted for ~6 months at each site, and may not reflect seasonal or annual variations in antimicrobial use. Patients had to pay for diagnostic testing and medical care at two of the hospitals, which may affect the type of patients admitted to the centers as well as the care delivered by physicians. Finally, all information was collected by prospective review of the medical record, thus data may be limited by the quality of the recorded information.

In conclusion, we systematically quantified the prevalence and indications for antimicrobial use at three tertiary care centers in LMICs. Despite being in disparate geographical locations and having different epidemiology of disease, we identified common potential targets for ASP interventions at these sites, including use of microbiologic culture data, partnership and consultation with microbiology/infectious diseases services, and appropriate diagnosis and treatment of LRTI. As initial steps in improving antimicrobial stewardship at these hospitals, we would suggest conducting collaborative sessions that include general medicine physicians and microbiology staff to discuss barriers to and methods for enhancing use of microbiologic culture data and consultation services. In addition, educational sessions regarding the appropriate diagnosis of common conditions such as LRTI, UTI, and skin/soft tissue infections could be conducted, with emphasis on the use of local guidelines and reminders regarding mechanisms to access these guidelines. Finally, antibiograms could be developed and implemented given the high use of empiric antimicrobials. Improving antimicrobial use in all settings will be important in the global strategy to tackle the growing threat of antimicrobial resistance.

## Data Availability Statement

The datasets presented in this article are not readily available because the datasets generated during the current study are not publicly available due to ethical reasons but may be available from the corresponding author on reasonable request and after conferring with the appropriate ethical review committees. Requests to access the datasets should be directed to LT, gayani.tillekeratne@duke.edu.

## Ethics Statement

Ethical approval for this study was obtained from the Kilimanjaro Christian Medical College Research Ethics and Review Committee and the National Institute for Medical Research (Tanzania), the Institutional Research and Ethics Committee of Moi University/Moi Teaching and Referral Hospital (Kenya), AMPATH (Kenya), the Ethical Review Committee of the Faculty of Medicine, University of Ruhuna (Sri Lanka), and the Duke University Institutional Review Board (United States). Written informed consent to participate in this study was provided by the participants' legal guardian/next of kin.

## Author Contributions

FM was involved in data collection oversight, writing the manuscript, and editing the manuscript. FM, CK, CB, AN, BP, RK, SA, and RM were involved in data collection oversight and editing the manuscript. JB was involved in writing and editing the manuscript. RR, AR, and TS were involved in data analysis and editing the manuscript. TØ, RD, PK, CW, and DA were involved in study design and editing the manuscript. BM was involved in study design, data collection oversight, and editing the manuscript. LT was responsible for overall study design and was involved in data collection, writing the manuscript, and editing the manuscript. All authors contributed to the article and approved the submitted version.

## Funding

This study was funded by a pilot grant from the Duke Global Health Institute. LT was supported by a grant from the National Institute of Allergy and Infectious Diseases (K23AI125677).

## Conflict of Interest

The authors declare that the research was conducted in the absence of any commercial or financial relationships that could be construed as a potential conflict of interest.

## Publisher's Note

All claims expressed in this article are solely those of the authors and do not necessarily represent those of their affiliated organizations, or those of the publisher, the editors and the reviewers. Any product that may be evaluated in this article, or claim that may be made by its manufacturer, is not guaranteed or endorsed by the publisher.
